# The interactive impact of social interaction and product involvement on customer stickiness in the context of live streaming e-commerce

**DOI:** 10.3389/fpsyg.2025.1724223

**Published:** 2026-01-12

**Authors:** Ligang Tian, Yilan Hai, Shulin Wang, Guang-bo Ma, Sudarshan Pillalamarri

**Affiliations:** 1 School of Economics and Management, Yingkou Institute of Technology, Yingkou, China; 2 Business School, Liaoning University, Shenyang, China; 3 Sunwah International Business School, Liaoning University, Shenyang, China; 4 Business and Law, De Montfort University The Gateway, Leicester, United Kingdom

**Keywords:** cognitive lock-in, customer trust, customer stickiness, live streaming e-commerce, product involvement, shopping motivation, social interaction

## Abstract

As live-streaming e-commerce markets mature, customer stickiness has become critical for platform success and a core prerequisite for its sustainable development. While social interaction serves as the primary engagement mechanism, its effectiveness varies across product types. This study examines the interactive effects of social interaction content and product involvement on customer stickiness in live-streaming e-commerce. Grounded in the stimulus-organism-response model and dual-process theory, three experiments with the total number of 1,360 participants used a 2 × 2 design manipulating social interaction type (information vs. relationship) and product involvement (high vs. low). Results reveal that information interaction generates higher customer stickiness under high involvement, while relationship interaction is more effective under low involvement. Customer trust and cognitive lock-in mediate these effects through emotional and cognitive pathways. Shopping motivation moderates these relationships. Specifically, utilitarian consumers benefit from both optimal combinations, while hedonic consumers only benefit from relationship interaction under low involvement. The findings provide evidence-based guidance for platforms to align interaction content with product characteristics and advance theoretical understanding of customer engagement mechanisms in live-streaming e-commerce.

## 1 Introduction

Live-streaming e-commerce has emerged as a transformative business model that reshapes consumer shopping behaviors and merchant marketing strategies worldwide. By integrating real-time video with interactive commerce, this format enables dynamic social interactions among hosts, viewers, and brands, distinguishing it from traditional e-commerce’s static product displays and delayed communication (Lin et al., 2021). The global market has experienced explosive growth. According to *the Live Commerce Platform Market report*, the global market size reached USD 12.8 billion in 2024 and is projected to grow at a compound annual growth rate of 18.2%, reaching USD 54.6 billion by 2033. China exemplifies this transformation: its market reached 4.92 trillion yuan in 2023 (a 40.48% year-on-year increase) with 597 million users. By May 2025, the China International Electronic Commerce Center reported that 72% of new customers are acquired through live-streaming channels. As this sector matures, competition has shifted from user acquisition to customer retention, making customer stickiness critical for platform viability (Lin et al., 2021; Wongkitrungrueng and Assarut, 2020; Bhattacharyya and Bose, 2020).

To capture this construct precisely, we distinguish between two dimensions: visit stickiness and purchase stickiness (Jiao et al., 2023; Bao and Zhu, 2023). Visit stickiness reflects sustained platform engagement, representing consumers’ long-term behavioral patterns. As a “traffic pool” for transaction conversion, it provides foundational strategic value for enterprises. Purchase stickiness, on the other hand, denotes recurring purchasing behavior, which signals consumers’ long-term purchase intention and directly contributes to growth in sales and profits (Jiao et al., 2023). Both dimensions are vital to platform sustainability, as they correspond to the visit–stay–purchase behavioral chain in live streaming. This distinction also prevents conflating ineffective stickiness patterns, such as high visit but low purchase, thereby enabling more targeted analysis of the factors driving different stages of consumer behavior.

Central to fostering both dimensions are social interaction, the core mechanism for information exchange and relationship building in live-streaming commerce (Allport et al., 2023). While prior studies have identified multiple antecedents of interaction, such as user characteristics (Kabacińska et al., 2025) and contextual features like gamification or KOL endorsements (Jiao et al., 2023), scholars increasingly emphasize that the content of interaction is a core driver of consumer behavioral outcomes. This study thus focuses on interaction content, adopting the dichotomy of informational interaction and relational interaction, which is theoretically rooted in communication theory and digital engagement research (Chuang and Yang, 2014; Zhou et al., 2019). Informational interaction transmits product details and utilitarian value, reducing cognitive uncertainty and facilitating rational decision-making. Relational interaction builds community, fosters shared identity, and establishes emotional affinity, shaping user attitudes and loyalty through socio-emotional connections. The effective deployment of these content strategies is key to sustaining the appeal of the socially co-created shopping experience.

However, the effectiveness of social interaction content varies based on product characteristics. Product involvement, the perceived personal relevance and decision risk associated with a product, fundamentally shapes how consumers process information (Dholakia, 2001). Extant research indicates that product involvement influences interaction effectiveness and consumer responses to various digital stimuli such as website gamification (Cowan and Ketron, 2019), and product captions (Candi et al., 2017). Yet the interactive effect between product involvement and social interaction content on stickiness remains theoretically underdeveloped.

This gap is critical. While social interaction and product involvement are often studied in isolation (Liu et al., 2018; Yin and Lee, 2024; Samala and Katkam, 2019; Mulcahy et al., 2020), Dual-process theory suggests their effects are contingent upon one another. Product involvement determines which cognitive processing system consumers activate, thereby influencing their responses to different interaction content (Evans and Stanovich, 2013).

Two additional gaps in the literature motivate this study. First, previous research has primarily adopted single-pathway perspectives when examining psychological mechanisms underlying customer stickiness, typically focusing on emotional processes while overlooking the multifaceted nature of consumer responses. This unidimensional approach fails to capture how social interactions and product characteristics jointly influence consumer psychology through both emotional and cognitive pathways (Hu et al., 2020; Wang et al., 2016). Second, prior research has predominantly explored environmental factors as boundary conditions (Kim et al., 2016), while the moderating role of individual consumer characteristics, particularly shopping motivation, remains empirically unvalidated.

To address these gaps, this study investigates three research questions: (1) How do informational versus relational interaction interact with product involvement to affect visit and purchase stickiness? (2) What are the mediating roles of customer trust and cognitive lock-in? (3) How does shopping motivation moderate these relationships? Our findings aim to provide a nuanced, theoretically grounded framework for developing context-sensitive interaction strategies in live-streaming e-commerce.

This research contributes to the literature in three ways. First, we provide systematic empirical evidence on how social interaction content and product involvement interact to influence customer stickiness, moving beyond previous research that examined these factors independently. Second, we identify and test key psychological mechanisms, customer trust and cognitive lock-in, through which these interactive effects operate, offering insights into both emotional and cognitive pathways. Third, we examine shopping motivation as a critical boundary condition, enhancing understanding of individual differences in response to social interaction strategies. Practically, our findings provide evidence-based guidance for platforms and merchants seeking to develop interaction strategies that align with product characteristics and consumer processing preferences.

## 2 Theoretical analysis and hypotheses development

### 2.1 Social interaction, product involvement, and customer stickiness

In live-streaming e-commerce, social interaction refers to the bidirectional information exchange and emotional communication among relevant social actors in the live broadcast scenario, encompassing both host-audience interaction and customer-customer interaction (Yadav and Pavlou, 2014). Host-audience interaction includes hosts’ product introductions, real-time replies to audience questions, and interactive activities. Customer-customer interaction involves audience comments, experience sharing, and mutual discussions in the live-stream chat room. Such social interactions generate feedback signals that shape consumer psychology and behavior (Xu, 2024).

Drawing upon the stimulus-organism-response (SOR) model, we conceptualize social interaction content as external stimuli (S) that trigger consumers’ cognitive and emotional states (O), ultimately influencing customer stickiness behaviors (R). The SOR framework is particularly suited to live-streaming contexts, where environmental stimuli, including host communication and peer interactions, continuously shape consumer experiences and behavioral responses (Huo et al., 2023). From a functional perspective, interactive platforms provide diverse communication channels that facilitate user feedback; from a perceptual perspective, such platforms effectively respond to users’ psychological needs; from an information exchange perspective, platform interactions establish feedback mechanisms between buyers and sellers, reducing information asymmetry and enhancing consumer trust.

However, the effectiveness of social interaction varies based on how consumers process interaction content. Dual-process theory explains this variation by proposing that individuals employ two distinct cognitive systems (Evans, 2008). The deliberative system engages in systematic, analytical, and effortful processing, while the intuitive system relies on automatic, heuristic-based responses. Critically, product involvement acts as a key trigger determining which system consumers activate, thereby shaping their processing of social interaction content (Evans and Stanovich, 2013; Lv et al., 2022; Ming et al., 2021).

Product involvement is closely associated with situational factors, product characteristics, and individual cognition (Dholakia, 2001). Under high product involvement, consumers engage in deliberate, analytical decision-making, devoting considerable effort to information search, alternative evaluation, and systematic comparison, thereby acquiring comprehensive knowledge of product and merchant characteristics prior to purchase. Conversely, under low product involvement, consumers engage in affect-driven decision-making with minimal information elaboration. They rely on peripheral cues, such as the welcoming atmosphere created by hosts to guide their purchase decisions (Fu et al., 2020).

Building on this theoretical foundation, we propose that the congruence between social interaction and consumer processing mode determines stickiness outcomes. Specifically, under high product involvement, consumers perceive greater decision stakes and uncertainty, activating deliberative processing characterized by extensive information search and systematic evaluation (Ruiz-Mafé et al., 2018). Informational interaction, conveying factual, analytical content about product specifications, features, and performance, aligns with this processing mode (Chuang and Yang, 2014). This congruence produces synergistic effects through two aspects. First, regarding visit stickiness, informational interaction enhances product transparency and reduces search costs, increasing consumer confidence in the platform’s evaluation support capabilities. When consumers find that a platform consistently provides comprehensive, reliable product information, they develop habitual reliance on the platform for future purchase decisions, thereby sustaining engagement (Mulcahy et al., 2020). Second, regarding purchase stickiness, access to detailed information decreases information asymmetry between consumers and merchants, building trust in merchant expertise and product quality. This trust reduces perceived purchase risk and increases willingness to conduct repeat transactions on the platform.

Under low product involvement, consumers perceive minimal decision consequences, activating intuitive processing that favors simplified decision rules and peripheral cues over extensive deliberation (Adhikari, 2019). Relational interaction, building emotional connections through shared experiences, empathetic responses, and community belonging, aligns with this processing mode (Zhou et al., 2019). This congruence also enhances customer stickiness. First, regarding visit stickiness, relational interaction creates positive emotional states and enhances social satisfaction, making the platform experience intrinsically enjoyable (Yahia et al., 2018). Consumers return to the platform not merely for transactions but for the social gratification and sense of belonging it provides. Second, regarding purchase stickiness, the emotional comfort and parasocial bonds developed through relational interaction reduce decision anxiety. When consumers feel emotionally connected to hosts or the community, they require less cognitive justification for purchases, facilitating spontaneous and repeat buying behavior (Kuenzel and Musters, 2007).

Therefore, we propose:

*H1a*: Social interaction and product involvement exhibit interactive effects on visit stickiness. Under high product involvement, information interaction leads to higher visit stickiness; under low product involvement, relationship interaction leads to higher visit stickiness.

*H1b*: Social interaction and product involvement exhibit interactive effects on purchase stickiness. Under high product involvement, information interaction leads to higher purchase stickiness; under low product involvement, relationship interaction leads to higher purchase stickiness.

### 2.2 Mediating role of customer trust

Within the SOR framework, customer trust represents a critical internal state (O) through which social interaction stimuli (S) influence customer stickiness behaviors (R). Customer trust is defined as consumers’ willingness to rely on an exchange partner based on confidence in the partner’s reliability, and benevolence (Özdemir and Sonmezay, 2020). In live-streaming e-commerce, customer trust reflects consumers’ confidence in hosts, merchants, and community members, encompassing beliefs about reliability, benevolence, and credibility that develop through social interactions (Song and Lin, 2024).

Research consistently demonstrates that social interaction features significantly promote customer trust development, which subsequently enhances continued usage intentions, customer engagement, and overall satisfaction (Li et al., 2023; Kumar et al., 2018). This trust-behavior linkage establishes customer trust as a critical mediating variable in the pathway linking social interaction to customer stickiness.

Under high product involvement, consumers engage in deliberative processing and actively seek evidence to evaluate merchant reliability and competence. Informational interaction builds customer trust in several ways. First, comprehensive and accurate product information demonstrates merchant expertise and knowledge, signaling competence and professionalism. Second, transparent communication about product specifications and honest comparisons reduces information asymmetry, indicating integrity and trustworthiness (Fu et al., 2022). When high-involvement consumers receive informational interaction that addresses their evaluation needs, they develop confidence in merchant capabilities and platform dependability, forming trust that reduces perceived risk and decision anxiety (Fulmer and Dirks, 2018).

Under low product involvement, consumers engage in intuitive processing and are more responsive to social and emotional cues rather than detailed information. Relational interaction builds customer trust through several ways. First, empathetic communication and emotional responsiveness demonstrate care and benevolence, fostering perceptions that merchants genuinely value consumer welfare. Second, shared experiences and community belonging create social bonds and interpersonal warmth that signal goodwill (Santos et al., 2021). When low-involvement consumers experience relational interaction that satisfies their social and emotional needs, they develop trust based on positive interpersonal experiences and perceived benevolence.

The trust developed through aligned interaction-involvement combinations subsequently enhances customer stickiness. Regarding visit stickiness, customer trust creates psychological comfort and security that motivates consumers to return to trusted platforms (Zheng et al., 2024; Hsieh, 2023). Regarding purchase stickiness, customer trust reduces perceived transaction risk and lowers psychological barriers to purchase decisions. When consumers trust merchants, they require less deliberation before committing to transactions, facilitating both initial and repeat purchases (Wang et al., 2022).

Therefore, we propose the following:

*H2a*: Customer trust mediates the interactive effects of social interaction and product involvement on visit stickiness. Aligned interaction-involvement combinations enhance visit stickiness through increased customer trust.

*H2b*: Customer trust mediates the interactive effects of social interaction and product involvement on purchase stickiness. Aligned interaction-involvement combinations enhance purchase stickiness through increased customer trust.

### 2.3 Mediating role of cognitive lock-in

Within the SOR framework, cognitive lock-in represents another critical internal state through which social interaction stimuli influence customer stickiness behaviors. Cognitive lock-in is defined as consumers’ reluctance to switch platforms or channels due to accumulated cognitive investments and learned usage patterns that create switching costs (Sénécal et al., 2015). In live-streaming e-commerce, cognitive lock-in reflects consumers’ development of platform-specific knowledge, usage expertise, and cognitive assets through continuous social interactions, encompassing investments in content understanding, feature mastery, and information processing capabilities (Murray and Häubl, 2007).

Research demonstrates that social interaction significantly promotes cognitive lock-in development, which subsequently enhances platform loyalty and continued usage (Shih, 2012; Ye et al., 2022). This establishes cognitive lock-in as a critical mediating variable linking social interaction to customer stickiness.

Under high product involvement, consumers engage in deliberative processing and actively seek comprehensive information. Informational interaction builds cognitive lock-in in several ways. First, detailed specifications and technical analyses require substantial effort to process, creating valuable platform-specific knowledge assets (Fu et al., 2020). Second, expert commentaries and comparative evaluations demand focused analytical reasoning, developing interpretive capabilities difficult to replicate elsewhere. When high-involvement consumers process informational interaction, they develop expertise and knowledge repositories tied to the platform, forming cognitive investments that would be costly to recreate elsewhere.

Under low product involvement, consumers engage in intuitive processing and respond more to social dynamics than analytical information. Relational interaction builds cognitive lock-in through two ways. First, understanding community norms and interaction conventions requires learning investments that create familiarity with platform-specific social environments (Liu et al., 2024). Second, accumulated knowledge of community culture and shared references creates platform-specific cognitive assets that become embedded in platform usage. When low-involvement consumers invest in understanding relational patterns, they develop social expertise that would require substantial effort to rebuild elsewhere (Lefebvre et al., 2016).

The cognitive lock-in developed through aligned combinations subsequently enhances customer stickiness. Higher cognitive lock-in increases perceived switching effort, making consumers more likely to continue using familiar platforms where they have made substantial investments, enhancing visit stickiness. Additionally, cognitive lock-in creates rational incentives to maximize returns on cognitive investments by completing transactions within platforms where consumers have developed expertise, enhancing purchase stickiness. Therefore, we propose:

*H3a*: Cognitive lock-in mediates the interactive effects of social interaction and product involvement on visit stickiness. Aligned interaction-involvement combinations enhance visit stickiness through increased cognitive lock-in.

*H3b*: Cognitive lock-in mediates the interactive effects of social interaction and product involvement on purchase stickiness. Aligned interaction-involvement combinations enhance purchase stickiness through increased cognitive lock-in.

### 2.4 Moderating role of shopping motivation

Shopping motivation represents a critical individual difference in goal orientation that guides consumers’ overall shopping behavior, independent of specific product attributes. Shopping motivation influences consumers’ content preferences, and evaluation criteria, creating systematic variations in how consumers respond to different interaction-involvement combinations (Qu et al., 2023). Consumer research distinguishes between utilitarian and hedonic shopping motivations (Childers et al., 2001). Utilitarian motivation treats shopping as a goal-directed task emphasizing efficiency and functionality, while hedonic motivation views shopping as an experiential process focused on enjoyment and emotional satisfaction (Kim and Kwon, 2025).

#### 2.4.1 Utilitarian-motivated consumers

For utilitarian-motivated consumers, social interaction effectiveness depends on facilitating efficient task completion. These consumers prioritize goal achievement and seek interactions that support rational decision-making.

Under high product involvement, utilitarian consumers engage in deliberative processing to make informed decisions. Informational interaction aligns with their efficiency goals by providing comprehensive product details and objective comparisons that reduce search costs and accelerate decision-making (Chang et al., 2023). In contrast, relational interaction introduces task-irrelevant social content that may distract from analytical processing and impede goal-directed evaluation.

Under low product involvement, utilitarian consumers maintain task orientation but rely on intuitive processing due to limited evaluation needs. Here, relational interaction becomes beneficial by providing social proof and normative cues that enable quick, confident decisions without extensive analysis (Scarpi, 2021). Conversely, informational interaction may impose unnecessary cognitive burden for low-stakes decisions, reducing efficiency.

#### 2.4.2 Hedonic-motivated consumers

For hedonic-motivated consumers, social interaction effectiveness depends on enhancing experiential satisfaction and shopping enjoyment. These consumers prioritize entertainment value and seek interactions that generate positive emotional experiences (An and Han, 2020).

Under low product involvement, hedonic consumers prioritize entertainment over analytical rigor. Relational interaction serves their experiential needs by fostering emotional connections, generating entertainment value, and creating engaging social experiences (Hu et al., 2018; Casaló et al., 2017). In contrast, informational interaction, though potentially informative, may trigger persuasion resistance and reduce experiential quality by imposing analytical demands that conflict with enjoyment-seeking goals.

Under high product involvement, hedonic consumers face conflicting demands between experiential preferences and analytical requirements. While they prefer relational interaction for its entertainment value, high-involvement decisions require substantial product evaluation that informational interaction better supports. This conflict neutralizes the differential effectiveness of interaction types. That is to say, neither interaction type fully satisfies both experiential and analytical demands, resulting in no significant difference between informational and relational interaction effects under high involvement conditions.

Based on the above analysis, we propose:

*H4a*: For utilitarian-motivated consumers, information interaction generates higher visit stickiness under high involvement, while relationship interaction generates higher visit stickiness under low involvement.

*H4b*: For utilitarian-motivated consumers, information interaction generates higher purchase stickiness under high involvement, while relationship interaction generates higher purchase stickiness under low involvement.

*H4c*: For hedonic-motivated consumers, relationship interaction generates higher visit stickiness under low involvement, with no significant difference between interaction types under high involvement.

*H4d*: For hedonic-motivated consumers, relationship interaction generates higher purchase stickiness under low involvement, with no significant difference between interaction types under high involvement.

Shopping motivation further moderates the mediating pathways through customer trust and cognitive lock-in. For utilitarian consumers, aligned interaction-involvement combinations facilitate goal achievement by building trust and cognitive lock-in. Specifically, informational interaction under high involvement and relational interaction under low involvement both support efficient task completion, thereby enhancing trust in platform reliability and increasing cognitive investments in platform-specific capabilities. For hedonic consumers, relational interaction under low involvement creates engaging experiences that strengthen emotional bonds and promote trust formation and cognitive lock-in development through enjoyable social learning processes. Therefore, we propose:

*H5a*: For utilitarian consumers, information interaction under high involvement and relationship interaction under low involvement generate higher visit stickiness through customer trust.

*H5b*: For hedonic consumers, relationship interaction under low involvement generates higher visit stickiness through customer trust, with no difference under high involvement.

*H5c*: For utilitarian consumers, information interaction under high involvement and relationship interaction under low involvement generate higher purchase stickiness through customer trust.

*H5d*: For hedonic consumers, relationship interaction under low involvement generates higher purchase stickiness through customer trust, with no difference under high involvement.

*H6a*: For utilitarian consumers, information interaction under high involvement and relationship interaction under low involvement generate higher visit stickiness through cognitive lock-in.

*H6b*: For hedonic consumers, relationship interaction under low involvement generates higher visit stickiness through cognitive lock-in, with no difference under high involvement.

*H6c*: For utilitarian consumers, information interaction under high involvement and relationship interaction under low involvement generate higher purchase stickiness through cognitive lock-in.

*H6d*: For hedonic consumers, relationship interaction under low involvement generates higher purchase stickiness through cognitive lock-in, with no difference under high involvement.

**Figure 1:**
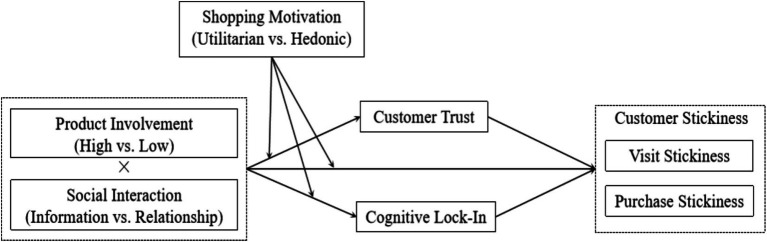
Theoretical framework.

Based on the hypotheses developed above, we construct the theoretical model (Figure 1) showing the relationships between social interaction, product involvement, customer trust, cognitive lock-in, customer stickiness, and shopping motivation.

## 3 Experimental design and results analysis

### 3.1 Pre-experiment: selection of experimental products

The pre-experiment aimed to identify appropriate experimental products by measuring participants’ familiarity with different products and assessing product involvement levels. The objective was to select products that were widely recognized and exhibited no significant gender differences in involvement perceptions.

Following previous research (Liu et al., 2013; Zhang and Wang, 2024) and consulting sales ranking data from major live-streaming platforms including Tmall, and TikTok, we initially selected 15 products for evaluation: cameras, electric bicycles, batteries, watches, laptops, jewelry, mobile phones, shampoo, electric toothbrushes, cookies, beverages, instant noodles, headphones, air conditioners, and jeans. These products were selected for their appropriateness as experimental stimuli. They represent major categories commonly featured in live-streaming e-commerce, spanning technology products, daily necessities, fashion items, and food products, ensuring ecological validity. Market data confirms their relevance. 3C products including mobile phones and headphones maintain substantial sales volume during 2025. Hair care products show 14.6% year-on-year growth, while apparel categories including jeans demonstrate consistent market presence. Importantly, participants’ familiarity with these commonly purchased products enables meaningful involvement assessments and authentic engagement with experimental scenarios.

A total of 109 college students (52 males, 57 females) were recruited to assess the involvement levels of these products using 7-point Likert scales. The results identified “mobile phones (*M* = 5.25),” “electric bicycles (*M* = 5.11),” and “laptops (*M* = 5.09)” as high involvement products, while “cookies (*M* = 2.81),” “jeans (*M* = 2.89),” and “headphones (*M* = 2.96)” were classified as low involvement products. No significant gender differences were found in mean scores across all selected products. Based on these findings, three experiments were designed with the following product pairings: Experiment 1 utilized “laptops” and “cookies,” Experiment 2 employed “mobile phones” and “jeans,” and Experiment 3 used “electric bicycles” and “headphones.” Each experiment paired one high-involvement product with one low-involvement product to test the hypothesized interaction effects.

### 3.2 Experiment 1: testing the interactive effects of social interaction and product involvement on customer stickiness

#### 3.2.1 Experimental purpose

Experiment 1 conducted a 2 (product involvement: high vs. low) × 2 (social interaction: information vs. relationship) between-groups experimental design to verify the interactive effects of social interaction and product involvement on customer stickiness. The experiment was conducted through the Credamo platform for questionnaire distribution. After screening for incomplete responses, 321 valid questionnaires were obtained, com-prising 152 males (47.35%) and 169 females (52.65%), with an average age of 26.8 years.

#### 3.2.2 Experimental materials and procedure

Following Zhang and Wang’s (2024) methodology, this study used textual scenarios to simulate live-streaming e-commerce environments. Experimental materials were developed based on authentic social interaction patterns observed in actual live-streaming contexts, categorized into two types. First, information interaction materials were derived from actual product specifications and focused on the host’s presentation and demonstration of product performance and quality attributes, supplemented by other viewers’ shared usage experiences. Second, relationship interaction materials centred on host-audience relational dynamics, emphasizing the host’s credibility and care for followers, accompanied by other viewers’ testimonials regarding positive communicative experiences with the host.

We randomly assigned participants to one of four experimental conditions, and instructed them to imagine they intended to purchase a laptop (or a box of cookies) and had entered the corresponding live-streaming room on an e-commerce platform. After reading the assigned experimental materials, participants completed measures of visit stickiness and purchase stickiness.

#### 3.2.3 Measurements

Social Interaction was measured using scales adapted from Zhou et al. (2019) and Liu et al. (2018). Information interaction comprised 4 items (e.g., “I can easily obtain product-related information through the host’s introduction”), while relationship interaction included 3 items (e.g., “Communication with other customers in the live-streaming room makes me feel happy”). Product Involvement was assessed using a 5-item scale adapted from Zaichkowsky (1994) and Mulcahy et al. (2020) (e.g., “When purchasing this type of product, you would carefully consider and comprehensively analyze product information”). Customer Stickiness was measured using scales from Jiao et al. (2023), with visit stickiness comprising 4 items (e.g., “I will visit this live-streaming room very frequently in the future”) and purchase stickiness comprising 4 items (e.g., “I will purchase products in this live-streaming room very frequently in the future”). All constructs were measured using 7-point Likert scales where 1 = strongly disagree and 7 = strongly agree. Demographic information was collected at the survey’s conclusion. To minimize response bias, item order was randomized across survey versions, and participants received compensation upon completion.

#### 3.2.4 Data analysis

*Manipulation checks*: The effectiveness of product involvement manipulation and social interaction manipulation were examined. First, Independent samples t-test results showed that the product involvement index for the high product involvement group (*M* = 5.234, SD = 1.270) was significantly higher than that of the low involvement group (*M* = 3.077, SD = 1.351), *t*(319) = 14.739, *p* < 0.001, 95% CI [1.869, 2.445]. This indicates that the product involvement manipulation was effective. Next, Independent samples t-test results showed that the relationship interaction index for the relationship interaction group (*M* = 5.031, SD = 0.895) was significantly higher than the information interaction index (*M* = 3.610, SD = 1.599), *t*(319) = 9.849, *p* < 0.001, 95% CI [1.137, 1.705]. The information interaction index for the information interaction group (*M* = 3.392, SD = 1.505) was significantly higher than the relationship interaction index (*M* = 1.977, SD = 0.727), *t*(319) = 10.765, *p* < 0.001, 95% CI [1.157, 1.674]. This indicates that the social interaction manipulation was effective.

*Interaction effects testing*: Two-way ANOVA was conducted with visit stickiness (Cronbach’s *α* = 0.893) and purchase stickiness (Cronbach’s *α* = 0.847) as dependent variables, and social interaction (relationship interaction = 0, information interaction = 1) and product involvement (low = 0, high = 1) as categorical independent variables. Results indicated that the main effect of social interaction on visit stickiness was not significant (*F*(1, 317) = 3.231, *p* > 0.05, *ƞ*_p_^2^ = 0.010), while its main effect on purchase stickiness was significant (F(1, 317) = 11.499, *p* < 0.01, *ƞ*_p_^2^ = 0.035). The main effect of product involvement was significant for both visit stickiness (*F*(1, 317) = 163.747, *p* < 0.001, *ƞ*_p_^2^ = 0.341) and purchase stickiness (*F*(1, 317) = 189.563, *p* < 0.001, *ƞ*_p_^2^ = 0.227). More relevant to the experimental interest, the interaction effect between social interaction and product involvement was significant for both visit stickiness (*F*(1, 317) = 52.481, *p* < 0.001, *ƞ*_p_^2^ = 0.142) and purchase stickiness (*F*(1, 317) = 43.628, *p* < 0.001, *ƞ*_p_^2^ = 0.121).

**Figure 2:**
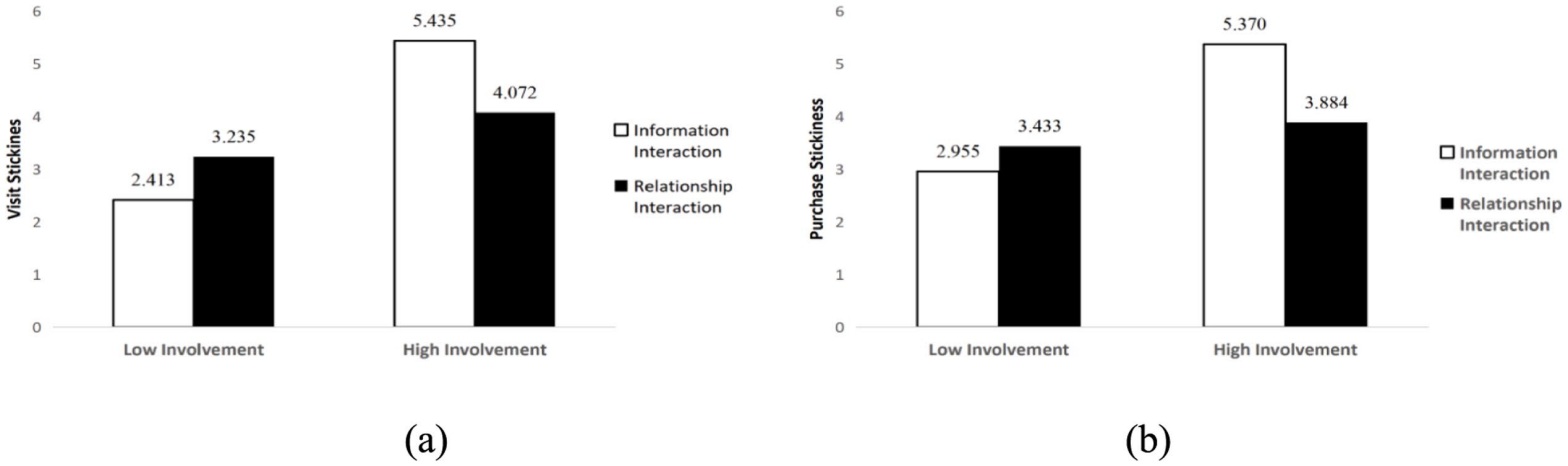
The interactive effects of social interaction and product involvement on customer stickiness: **(a)** Visit stickiness; **(b)** Purchase stickiness.

Further analysis revealed that under high product involvement conditions, information interaction led to higher visit stickiness (*M*_information_ = 5.435, SD = 0.150; *M*_relationship_ = 4.072, SD = 0.151, *p* < 0.001, 95% CI [0.944, 1.782]) and purchase stickiness (*M*_information_ = 5.370, SD = 0.148; *M*_relationship_ = 3.884, SD = 0.149, *p* < 0.05, 95% CI [1.073, 1.899]). Under low product involvement conditions, relationship interaction led to higher visit stickiness (*M*_relationship_ = 3.235, SD = 0.149; *M*_information_ = 2.413, SD = 0.153, *p* < 0.001, 95% CI [0.401, 1.242]) and purchase stickiness (*M*_relationship_ = 3.433, SD = 0.147; *M*_information_ = 2.955, SD = 0.151, *p* < 0.05, 95% CI [0.063, 0.892]). H1a and H1b were supported, with specific test results shown in Figure 2.

### 3.3 Experiment 2: testing the mediating effects of customer trust and cognitive lock-in

#### 3.3.1 Experimental purpose

Experiment 2 examined the mediating mechanisms underlying the interactive effects of social interaction and product involvement on customer stickiness. This experiment tested the mediating roles of customer trust (H2a, H2b) and cognitive lock-in (H3a, H3b) while replicating the direct interactive effects from Experiment 1 (H1a, H1b) to enhance robustness. The experiment employed a 2 (product involvement: high vs. low) × 2 (social interaction: information vs. relationship) between-groups experimental design. Data col-lection was conducted online through the Credamo platform. After excluding invalid responses, 423 valid questionnaires were retained, comprising 209 males (49.41%) and 214 females (50.59%) with a mean age of 27.6 years.

#### 3.3.2 Experimental procedure and measurement

Experiment 2 followed the same operational procedure as Experiment 1, with two key additions: customer trust and cognitive lock-in measurements. To enhance the relibility of research conclusions, the stimulus products were changed to “mobile phones” and “jeans.” Customer trust measurement was adapted from scales developed by Fulmer and Dirks (2018), comprising 3 items (e.g., “When watching live streams, I believe that the products provided by merchants have quality assurance”). Cognitive lock-in measurement was adapted from scales developed by Shi et al. (2018), comprising 4 items (e.g., “When watching live streams, I prioritize visiting followed live-streaming rooms and choose to purchase from them, even when I know they may not be the best option”). Like Experiment 1, participants in Experiment 2 were randomly assigned to one of four experimental groups. After reading the respective experimental materials, participants completed measurements of customer trust, cognitive lock-in, visit stickiness, and purchase stickiness. The manipulation of product involvement and social interaction, as well as the measurement of customer stickiness, remained identical to Experiment 1.

#### 3.3.3 Data analysis

*Manipulation checks*: The effectiveness of product involvement manipulation and social interaction manipulation were examined. Independent samples t-test results showed that the product involvement index for the high product involvement group (*M* = 5.616, SD = 1.435) was significantly higher than that of the low involvement group (*M* = 3.881, SD = 1.146), *t*(421) = 13.732, *p* < 0.001, 95% CI [1.487, 1.983]. This indicates that the product involvement manipulation was effective. Meanwhile, Independent samples t-test results showed that the relationship interaction index for the relationship interaction group (*M* = 4.714, SD = 1.502) was significantly higher than the information interaction index (*M* = 3.543, SD = 1.645), *t*(421) = 7.642, *p* < 0.001, 95% CI [0.869, 1.471]. The information interaction index for the information interaction group (*M* = 4.780, SD = 1.407) was significantly higher than the relationship interaction index (*M* = 3.425, SD = 1.580), *t*(421) = 9.314, *p* < 0.001, 95% CI [1.069, 1.641]. This indicates that the social interaction manipulation was effective.

*Interaction effects testing*: Two-way ANOVA was conducted with visit stickiness (Cronbach’s *α* = 0.883) and purchase stickiness (Cronbach’s *α* = 0.879) as dependent variables, and social interaction (relationship interaction = 0, information interaction = 1) and product involvement (low = 0, high = 1) as categorical independent variables. Results indicated that the main effects of social interaction on visit stickiness (*F*(1, 419) = 30.147, *p* < 0.001, *ƞ*_p_^2^ = 0.067) and purchase stickiness (*F*(1, 419) = 33.696, *p* < 0.001, *ƞ*_p_^2^ = 0.074) were significant. The main effects of product involvement were significant for both visit stickiness (*F*(1, 419) = 208.085, *p* < 0.001, *ƞ*_p_^2^ = 0.332) and purchase stickiness (*F*(1, 419) = 183.078, *p* < 0.001, *ƞ*_p_^2^ = 0.304). More importantly, the interaction effects between social interaction and product involvement were significant for both visit stickiness (*F*(1, 419) = 93.837, *p* < 0.001, *ƞ*_p_^2^ = 0.183) and purchase stickiness (*F*(1, 419) = 75.020, *p* < 0.001, *ƞ*_p_^2^ = 0.152).

**Figure 3:**
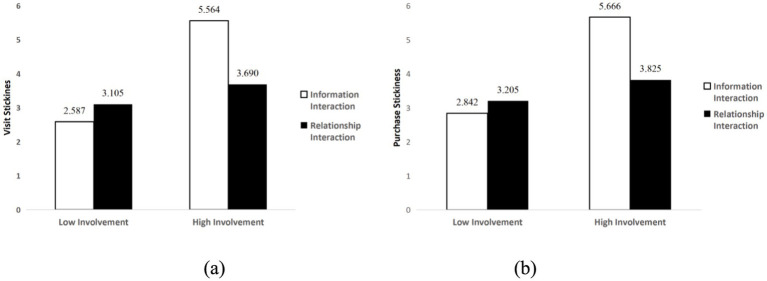
The interactive effects of social interaction and product involvement on customer stickiness: **(a)** Visit stickiness; **(b)** Purchase stickiness.

Further analysis revealed that under high product involvement conditions, information interaction led to higher visit stickiness (*M*_information_ = 5.564, SD = 0.119; *M*_relationship_ = 3.690, SD = 0.127, *p* < 0.001, 95% CI [1.532, 2.216]) and purchase stickiness (*M*_information_ = 5.666, SD = 0.123; *M*_relationship_ = 3.825, SD = 0.131, *p* < 0.001, 95% CI [1.488, 2.193]). Under low product involvement conditions, relationship interaction led to higher visit stickiness (*M*_relationship_ = 3.105, SD = 0.120; *M*_information_ = 2.587, SD = 0.128, *p* < 0.01, 95% CI [0.174, 0.863]) and purchase stickiness (*M*_relationship_ = 3.205, SD = 0.123; *M*_information_ = 2.842, SD = 0.132, *p* < 0.05, 95% CI [0.008, 0.719]). Hypotheses H1a and H1b were again supported, with results shown in Figure 3.

*Mediation effects testing*: Using social interaction as the independent variable, visit stickiness (purchase stickiness) as the dependent variable, customer trust (Cronbach’s *α* = 0.938) and cognitive lock-in (Cronbach’s *α* = 0.960) as mediating variables, and product involvement as the moderating variable, Process Model 8 was employed with the Boot-strap method to test the mediation effects of customer trust and cognitive lock-in. The confidence level was set at 95% with a sample size of 5,000. Results showed that the mediation effect coefficients of customer trust were 0.568 (BootSE = 0.138, 95% CI [0.309, 0.847]) and 0.635 (BootSE = 0.145, 95% CI [0.360, 0.927]) respectively, with confidence intervals not containing 0, indicating significant mediation effects. Specifically, as shown in Table 1, under high product involvement conditions, customer trust mediated the positive effects of social interaction on visit stickiness and purchase stickiness, with mediation effect coefficients of 0.361 (BootSE = 0.088, 95% CI [0.195, 0.536]) and 0.404 (BootSE = 0.090, 95% CI [0.231, 0.586]) respectively, with confidence intervals not containing 0. These results indicate that under high product involvement conditions, compared to relationship interaction, information interaction generates higher customer trust, thereby producing stronger visit stickiness and purchase stickiness. Under low product involvement conditions, customer trust played a negative mediating role in the positive effects of social interaction on visit stickiness and purchase stickiness, with mediation effect coefficients of −0.207 (BootSE = 0.062, 95% CI [−0.339, −0.099]) and −0.231 (BootSE = 0.068, 95% CI [−0.379, −0.112]) respectively, with confidence intervals not containing 0. These results indicate that under low product involvement conditions, compared to information interaction, relationship interaction generates higher customer trust, thereby producing stronger visit stickiness and purchase stickiness. Thereby, H2a and H2b were supported.

**Table 1:** Mediating effects test results.

Path	High product involvement			Low product involvement		
	Effect	SE	95%CI	Effect	SE	95%CI
SI → CT → VS	0.361	0.088	[0.195, 0.536]	−0.207	0.062	[−0.339, −0.099]
SI → CT → PS	0.404	0.090	[0.231, 0.586]	−0.231	0.068	[−0.379, −0.112]
SI → CL → VS	0.201	0.062	[0.091, 0.331]	−0.095	0.045	[−0.195, −0.023]
SI → CL → PS	0.182	0.061	[0.070, 0.310]	−0.087	0.042	[−0.180, −0.018]

SI, social interaction; CT, customer trust; CL, cognitive lock-in; VS, visit stickiness; and PS, purchase stickiness. (The same abbreviations apply throughout the following tables).

Similarly, the mediation effect coefficients of cognitive lock-in were 0.296 (BootSE = 0.092, 95% CI [0.135, 0.493]) and 0.269 (BootSE = 0.091, 95% CI [0.104, 0.458]) respectively, with confidence intervals not containing 0, indicating significant mediation effects. Specifically, as shown in Table 1, under high product involvement conditions, cognitive lock-in mediated the positive effects of social interaction on visit stickiness and purchase stickiness, with mediation effect coefficients of 0.201 (BootSE = 0.062, 95% CI [0.091, 0.331]) and 0.182 (BootSE = 0.061, 95% CI [0.070, 0.310]) respectively, with confidence intervals not containing 0. These results indicate that under high product involvement conditions, compared to relationship interaction, information interaction generates higher cognitive lock-in, thereby producing stronger visit stickiness and purchase stickiness. Under low product involvement conditions, cognitive lock-in played a negative mediating role in the positive effects of social interaction on visit stickiness and purchase stickiness, with mediation effect coefficients of −0.095 (BootSE = 0.045, 95% CI [−0.195, −0.023]) and −0.087 (BootSE = 0.042, 95% CI [−0.180, −0.018]) respectively. These results indicate that under low product involvement conditions, compared to information interaction, relationship interaction generates higher cognitive lock-in, thereby producing stronger visit stickiness and purchase stickiness. Hypotheses H3a and H3b were supported.

### 3.4 Experiment 3: testing the moderating effects of shopping motivation

#### 3.4.1 Experimental purpose

Experiment 3 also employed the 2 (product involvement: high vs. low) × 2 (social interaction: information vs. relationship) experimental design to verify the moderating role of shopping motivation in the customer stickiness formation process within live-streaming e-commerce contexts. The experiment was conducted through the Credamo platform for questionnaire distribution. After excluding invalid responses, 507 valid questionnaires were obtained, comprising 236 males (46.55%) and 271 females (53.45%), with a mean age of 26.2 years.

#### 3.4.2 Experimental procedure and measurement

Experiment 3 incorporated the measurement of shopping motivation as a moderating variable. Shopping motivation represents the internal driving force of consumer behavior, arising from consumer needs and interests in relation to the shopping environment (Deci and Ryan, 1985; Pappas et al., 2017). This study adapted scales developed by Büttner et al. (2014) and Voss et al. (2003) to measure shopping motivation, including 3 items for utilitarian motivation and 4 items for hedonic motivation. Participants completed all scale items, with average scores computed for each motivation dimension to represent their respective levels. To ensure sufficient variability in individual shopping motivation profiles, only participants with distinct scores across the two motivation dimensions were included in the final sample. Specifically, respondents whose utilitarian and hedonic motivation scores were simultaneously high or low were excluded to eliminate potential confounding effects associated with such uniformity. Participants’ shopping motivation type was determined by comparing their average scores on the two dimensions, and this categorical variable was subsequently coded with hedonic = 0, utilitarian = 1. All experimental products selected in this study exhibited both hedonic and utilitarian characteristics. Thus, measuring shopping motivation ensured that differences in shopping motivation originated from individual consumers rather than being influenced by product attributes.

To enhance the reliability of research results, Experiment 3 changed the stimulus products to “electric bicycles” and “headphones.” Like Experiments 1 and 2, participants in Experiment 3 were randomly assigned to one of four experimental groups. After reading the respective experimental materials, participants completed measurements of shopping motivation, customer trust, cognitive lock-in, and customer stickiness. The manipulation of product involvement and social interaction, as well as the measurement of customer trust, cognitive lock-in, and customer stickiness in Experiment 3, remained identical to Experiment 2.

#### 3.4.3 Data analysis

*Manipulation checks*: The effectiveness of product involvement manipulation and social interaction manipulation were examined. Independent samples t-test results showed that the product involvement index for the high product involvement group (*M* = 5.032, SD = 1.583) was significantly higher than that of the low product involvement group (*M* = 3.701, SD = 1.482), *t*(505) = 9.710, *p* < 0.001, 95% CI [1.056, 1.592]. This indicates that the product involvement manipulation was effective. Independent samples t-test results showed that the relationship interaction index for the relationship interaction group (*M* = 4.688, SD = 1.498) was significantly higher than the information interaction index (*M* = 3.598, SD = 1.668), *t*(505) = 7.751, *p* < 0.001, 95% CI [0.814, 1.366]. The information interaction index for the information interaction group (*M* = 4.786, SD = 1.404) was significantly higher than the relationship interaction index (*M* = 3.484, SD = 1.579), *t*(505) = 9.753, *p* < 0.001, 95% CI [1.039, 1.562]. This indicates that the social interaction manipulation was effective.

*Moderation effects testing*: Three-way ANOVA was conducted with visit stickiness (Cronbach’s *α* = 0.900) and purchase stickiness (Cronbach’s *α* = 0.891) as dependent variables, and social interaction (relationship interaction = 0, information interaction = 1), product involvement (low = 0, high = 1), and shopping motivation (hedonic = 0, utilitarian = 1) as categorical independent variables. Results indicated that the main effects of social interaction were significant for both visit stickiness (*F*(1, 499) = 6.938, *p* < 0.01, *ƞ*_p_^2^ = 0.014) and purchase stickiness (*F*(1, 499) = 4.159, *p* < 0.05, *ƞ*_p_^2^ = 0.008). The main effects of product involvement were significant (*F*(1, 499) = 140.618, *p* < 0.001, *ƞ*_p_^2^ = 0.220; F(1, 499) = 168.878, *p* < 0.001, *ƞ*_p_^2^ = 0.253). The three-way interaction effects were significant for both visit stickiness (*F*(1, 499) = 55.410, *p* < 0.001, *ƞ*_p_^2^ = 0.100) and purchase stickiness (*F*(1, 499) = 28.709, *p* < 0.001, *ƞ*_p_^2^ = 0.054).

Further interaction effects analysis revealed that for utilitarian motivation consumers, the interaction effects between social interaction and product involvement were significant (*F*(1, 228) = 190.906, *p* < 0.001, *ƞ*_p_^2^ = 0.456; F(1, 228) = 122.849, *p* < 0.001, *ƞ*_p_^2^ = 0.350). Under high product involvement conditions, information interaction led to higher visit stickiness (*M*_information_ = 6.703, SD = 0.181; *M*_relationship_ = 3.539, SD = 0.150, *p* < 0.001, 95% CI [2.701, 3.627]) and purchase stickiness (*M*_information_ = 6.729, SD = 0.183; *M*_relationship_ = 4.268, SD = 0.152, *p* < 0.001, 95% CI [1.993, 2.930]). Under low product involvement conditions, relationship interaction led to higher visit stickiness (*M*_relationship_ = 3.925, SD = 0.166; *M*_information_ = 2.500, SD = 0.166, *p* < 0.001, 95% CI [0.963, 1.888]) and purchase stickiness (*M*_relationship_ = 3.759, SD = 0.168; *M*_information_ = 2.496, SD = 0.168, *p* < 0.001, 95% CI [0.795, 1.731]). Hypotheses H4a and H4b were supported.

**Figure 4:**
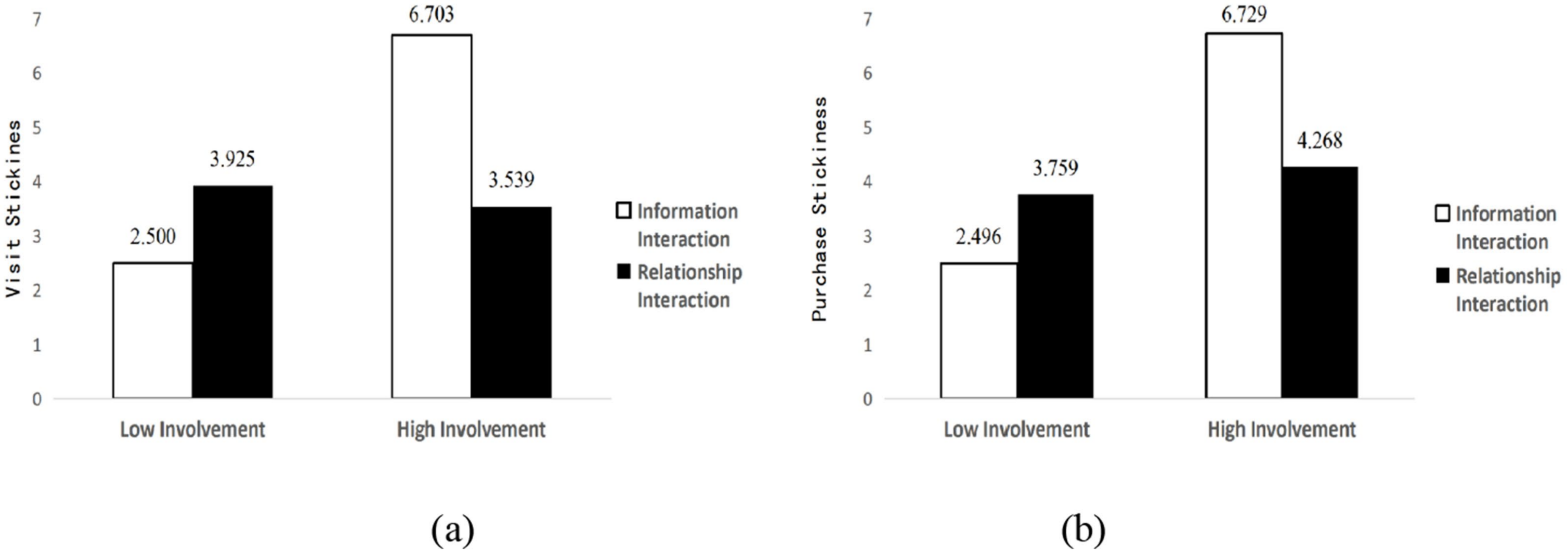
Moderating effect of shopping motivation (utilitarian motivation): **(a)** Visit stickiness; **(b)** Purchase stickiness.

**Figure 5:**
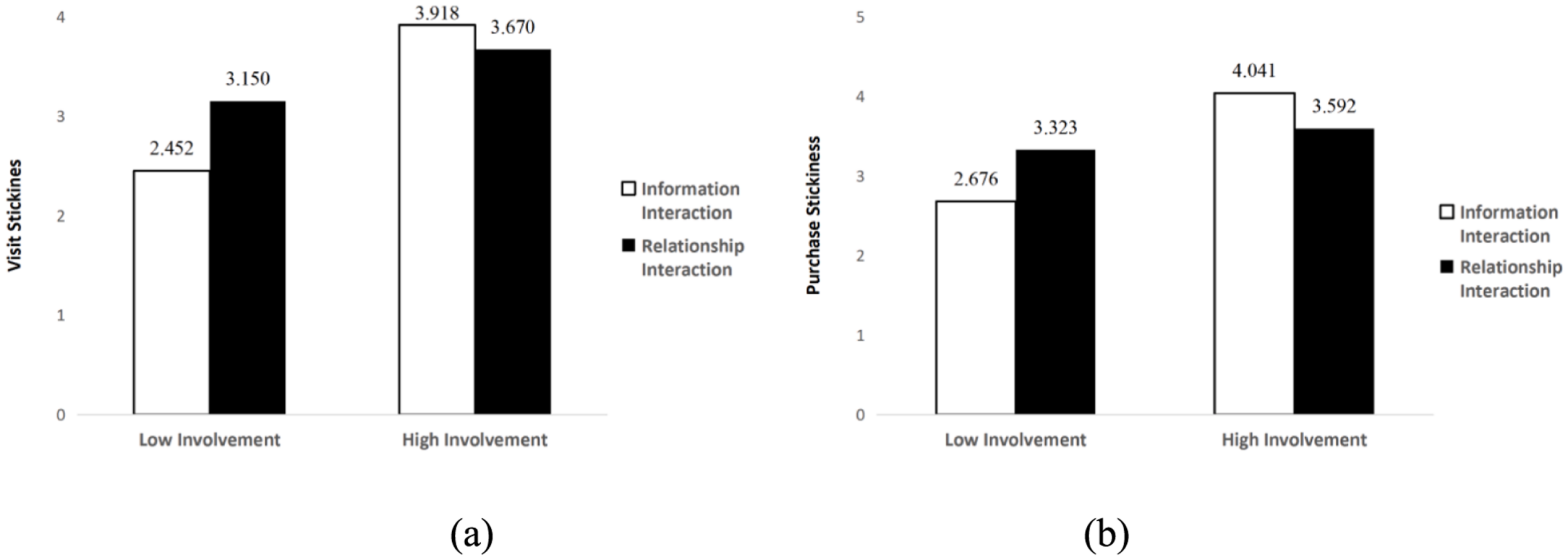
Moderating effect of shopping motivation (hedonic motivation): **(a)** Visit stickiness; **(b)** Purchase stickiness.

For hedonic motivation consumers, the interaction effects between social interaction and product involvement were significant for both visit stickiness (*F*(1, 271) = 7.217, *p* < 0.01, *ƞ*_p_^2^ = 0.026) and purchase stickiness (*F*(1, 271) = 9.722, *p* < 0.01, *ƞ*_p_^2^ = 0.035). Under low product involvement conditions, relationship interaction led to higher visit stickiness (*M*_relationship_ = 3.150, SD = 0.193; *M*_information_ = 2.452, SD = 0.162, *p* < 0.01, 95% CI [0.202, 1.194]) and purchase stickiness (*M*_relationship_ = 3.323, SD = 0.192; *M*_information_ = 2.676, SD = 0.161, *p* < 0.05, 95% CI [0.152, 1.141]). However, under high product involvement conditions, there were no significant differences between information interaction and relationship interaction for visit stickiness (*M*_information_ = 3.918, SD = 0.193; *M*_relationship_ = 3.670, SD = 0.153, *p* > 0.05, 95% CI [−0.237, 0.734]) and purchase stickiness (*M*_information_ = 4.041, SD = 0.192; *M*_relationship_ = 3.592, SD = 0.153, *p* > 0.05, 95% CI [−0.035, 0.933]). Hypotheses H4c and H4d were supported, with results shown in Figures 4 and 5.

*Moderated mediation effects testing*: Using social interaction as the independent variable, visit stickiness (purchase stickiness) as the dependent variable, customer trust (Cronbach’s *α* = 0.944) and cognitive lock-in (Cronbach’s α = 0.955) as mediating variables, and product involvement and shopping motivation as moderating variables, Process Model 12 was employed with the Bootstrap method to test the mediation effects of customer trust and cognitive lock-in. The confidence level was set at 95% with a sample size of 5,000. Results showed that the moderated mediation effect coefficients of customer trust were 0.153 (BootSE = 0.087, 95% CI [0.017, 0.350]) and 0.185 (BootSE = 0.098, 95% CI [0.025, 0.406]), indicating significant moderated mediation effects.

Specifically, as shown in Table 2, for utilitarian motivation consumers under high product involvement conditions, customer trust mediated the positive effects of social interaction on visit stickiness and purchase stickiness, with mediation effect coefficients of 0.309 (BootSE = 0.110, 95% CI [0.096, 0.523]) and 0.372 (BootSE = 0.107, 95% CI [0.171, 0.593]), with confidence intervals not containing 0. These results indicate that under high product involvement conditions, compared to relationship interaction, information interaction generates higher customer trust, thereby producing stronger visit stickiness and purchase stickiness. Under low product involvement conditions, customer trust played a negative mediating role in the positive effects of social interaction on visit stickiness and purchase stickiness, with mediation effect coefficients of −0.068 (BootSE = 0.044, 95% CI [−0.168, −0.002]) and −0.081 (BootSE = 0.051, 95% CI [−0.200, −0.001]). These results indicate that under low product involvement conditions, compared to information inter-action, relationship interaction generates higher customer trust, thereby producing stronger visit stickiness and purchase stickiness. Hypotheses H5a and H5c were supported.

**Table 2:** Moderated mediation effect test results.

Shopping motivation	Path	High product involvement			Low product involvement		
		Effect	SE	95%CI	Effect	SE	95%CI
Utilitarian motivation	SI → CT → VS	0.309	0.110	[0.096, 0.523]	−0.068	0.044	[−0.168, −0.002]
	SI → CT → PS	0.372	0.107	[0.171, 0.593]	−0.081	0.051	[−0.200, −0.001]
	SI → CL → VS	0.618	0.094	[0.446, 0.806]	−0.383	0.101	[−0.589, −0.191]
	SI → CL → PS	0.409	0.090	[0.235, 0.588]	−0.254	0.079	[−0.420, −0.114]
Hedonic motivation	SI → CT → VS	0.159	0.063	[−0.045, 0.293]	−0.064	0.039	[−0.152, −0.002]
	SI → CT → PS	0.192	0.166	[−0.078, 0.334]	−0.077	0.045	[−0.181, −0.007]
	SI → CL → VS	0.371	0.090	[−0.202, 0.554]	−0.171	0.082	[−0.332, −0.011]
	SI → CL → PS	0.246	0.071	[−0.119, 0.397]	−0.113	0.062	[−0.248, −0.006]

For hedonic motivation consumers, under low product involvement conditions, customer trust played a negative mediating role in the positive effects of social interaction on visit stickiness and purchase stickiness, with mediation effect coefficients of −0.064 (BootSE = 0.039, 95% CI [−0.152, −0.002]) and −0.077 (BootSE = 0.045, 95% CI [−0.181, −0.007]) respectively. These results indicate that under low product involvement conditions, compared to information interaction, relationship interaction generates higher customer trust, thereby producing stronger visit stickiness and purchase stickiness. Under high product involvement conditions, customer trust did not play a mediating role in the effects of social interaction on visit stickiness and purchase stickiness (95% CI [−0.045, 0.293]; 95% CI [−0.078, 0.334]), with confidence intervals containing 0. Hypotheses H5b and H5d were supported.

The mediation effect coefficients of cognitive lock-in were 0.459 (BootSE = 0.167, 95% CI [0.160, 0.807]) and 0.304 (BootSE = 0.120, 95% CI [0.088, 0.559]), with confidence intervals not containing 0, indicating significant moderated mediation effects. Specifically, as shown in Table 2, for utilitarian motivation consumers under high product involvement conditions, cognitive lock-in mediated the positive effects of social interaction on visit stickiness and purchase stickiness, with mediation effect coefficients of 0.618 (BootSE = 0.094, 95% CI [0.446, 0.806]) and 0.409 (BootSE = 0.090, 95% CI [0.235, 0.588]) respectively. These results indicate that under high product involvement conditions, compared to relationship interaction, information interaction generates higher cognitive lock-in, thereby producing stronger visit stickiness and purchase stickiness. Under low product involvement conditions, cognitive lock-in played a negative mediating role in the positive effects of social interaction on visit stickiness and purchase stickiness, with mediation effect coefficients of −0.383 (BootSE = 0.101, 95% CI [−0.589, −0.191]) and −0.254 (BootSE = 0.079, 95% CI [−0.420, −0.114]) respectively. These results indicate that under low product involvement conditions, compared to information interaction, relationship interaction generates higher cognitive lock-in, thereby producing stronger customer stickiness. Hypotheses H6a and H6c were supported.

For hedonic motivation consumers, under low product involvement conditions, cognitive lock-in played a negative mediating role in the positive effects of social interaction on visit stickiness and purchase stickiness, with mediation effect coefficients of −0.171 (BootSE = 0.082, 95% CI [−0.332, −0.011]) and −0.113 (BootSE = 0.062, 95% CI [−0.248, −0.006]) respectively. These results indicate that under low product involvement conditions, compared to information interaction, relationship interaction generates higher cognitive lock-in, thereby producing stronger customer stickiness. Under high product involvement conditions, cognitive lock-in did not play a mediating role in the positive effects of social interaction on visit stickiness and purchase stickiness (95% CI [−0.202, 0.554]; 95% CI [−0.119, 0.397]), with confidence intervals containing 0. Hypotheses H6b and H6d were supported.

## 4 Discussion

### 4.1 Research results

Based on the live-streaming e-commerce context and grounded in the SOR model and dual-process theory, this study examined the interactive effects of social interaction and product involvement on customer stickiness through three experiments. The following key findings were obtained:

First, social interaction effects on customer stickiness are contingent upon product involvement levels. Results demonstrate a significant interaction effect where information interaction generates higher customer stickiness under high product involvement conditions, while relationship interaction produces higher customer stickiness under low product involvement conditions. This pattern reflects the alignment between interaction types and consumers’ information processing modes. Deliberative processing systems favor information-rich interactions for high-involvement decisions, while intuitive processing systems favor relationship-based interactions for low-involvement decisions.

Second, social interaction and product involvement influence customer stickiness formation through both emotional and cognitive pathways. Information interaction under high product involvement conditions and relationship interaction under low product involvement conditions enhance customer trust and cognitive lock-in, thereby improving customer stickiness. Dual-pathway mediation mechanisms explain how social interaction and product involvement influence customer stickiness. The study reveals that both customer trust and cognitive lock-in mediate the interactive effects. Specifically, information interaction under high involvement and relationship interaction under low involvement enhance both customer trust and cognitive lock-in, which subsequently increase visit and purchase stickiness.

Third, shopping motivation serves as a critical boundary condition. For utilitarian-motivated consumers, both optimal interaction-involvement combinations effectively enhance customer trust and cognitive lock-in, generating strong customer stickiness. For hedonic-motivated consumers, only relationship interaction under low involvement proves effective, while high involvement conditions neutralize interaction type differences due to conflicts between experiential preferences and analytical demands.

### 4.2 Theoretical implications

First, these findings advance social interaction theory by identifying product involvement as a critical contingency factor. Observed interaction effects support the notion that social interaction effectiveness depends on alignment with consumers’ information-processing styles. This implies social interaction theory should incorporate dual-process frameworks, moving beyond universal effect assumptions to contingency-based models that account for situational factors in predicting sustained customer stickiness. This echoes prior research on the interaction between product involvement and interactive factors (Zhang and Wang, 2024), extending social interaction’s impact on consumer behavior to the long-term domain, broadening its research scope in consumer behavior studies.

Second, confirmed dual mediation validates parallel pathway models of customer stickiness. The concurrent operation of emotional (i.e., customer trust) and cognitive (i.e., cognitive lock-in) pathways underscores the need for comprehensive stickiness models to integrate both affective and cognitive mechanisms, rather than treating them as mutually exclusive. While prior studies established customer trust’s positive effect on stickiness (Wang et al., 2016), they overlooked cognitive lock-in’s role in its formation. This study introduces the lock-in effect into the field of marketing research. Via a parallel mediation model linking social interaction, product involvement, and customer stickiness, it clarifies how social interaction shapes stickiness from emotional and cognitive perspectives, thus expanding the application of these two mediating variables in marketing studies.

Third, the moderating role of shopping motivation highlights the theoretical significance of individual differences in consumer behavior. Specifically, utilitarian and hedonic motivations elicit heterogeneous responses to social interaction and product involvement, underscoring that motivation-based segmentation is central to stickiness theory, which aligns with the insights from Putri and Setyawan (2022) and Chang et al. (2023). By empirically validating shopping motivation as a critical boundary condition, this study enriches the theoretical landscape of consumer stickiness research.

### 4.3 Management implications

First, live-streaming platforms should implement involvement-based interaction strategies. For high-involvement products, platforms should prioritize information-rich content including detailed product demonstrations, technical specifications, and expert comparisons. For low-involvement products, emphasis should be placed on relationship-building content such as host-audience interactions, community building, and entertainment value to enhance emotional connections.

Second, platforms should develop dual-pathway customer retention strategies. Understanding that customer stickiness operates through both emotional and cognitive mechanisms, platforms should simultaneously build customer trust through authentic interactions and transparent practices while creating cognitive lock-in through personalized recommendations, switching costs, and platform-specific benefits that increase de-pendency.

Third, platforms should implement motivation-based customer segmentation. Utilitarian consumers should receive consistent, goal-oriented content regardless of product involvement, while hedonic consumers require careful content curation that emphasizes experiential value, particularly for low-involvement products. For high-involvement products, hedonic consumers may benefit from hybrid approaches that balance analytical content with entertaining presentation formats.

### 4.4 Limitations and future research

This study has several limitations that suggest directions for future research. First, the reliance on scenario-based experiments, while methodologically sound, may not fully capture the complexity of actual live-streaming environments. Future research should examine real shopping behaviors through field experiments or analysis of actual consumer data from live-streaming platforms to enhance ecological validity. Second, this study only examined the mediating roles of customer trust and cognitive lock-in. Future research should explore additional mediating variables such as processing fluency, customer engagement to develop more comprehensive theoretical models. Finally, experimental participants were mainly limited to young adults, lacking broader demographic data support. Future research could consider supplementing experimental data from more groups to make research coverage more comprehensive.

## Data Availability

The original contributions presented in the study are included in the article/Supplementary material, further inquiries can be directed to the corresponding author/s.
