# Supplementary material for: The interactive impact of social interaction and product involvement on customer stickiness in the context of live streaming e-commerce
Source: Front Psychol. 2026 Jan 12;16:1724223. doi: 10.3389/fpsyg.2025.1724223 (PMC12833047; doi:10.3389/fpsyg.2025.1724223)
Supplement: Supplementary file 1 [file Supplementary_file_1.docx]

Supplementary Material

# Appendix A. Experiment Material

Hello, thank you for participating in this research study on consumer behavior in live-streaming e-commerce. All responses will be anonymized and utilized exclusively for academic research, ensuring complete privacy protection. Please carefully read and respond to each item as instructed. There are no correct or incorrect responses. Upon completion, you may contact the research team to receive compensation. If you agree to proceed, please click "Continue." Thank you for your support and cooperation!

**Part I: Scenario Introduction**

Imagine that on a Saturday evening at 8:00 PM, you enter a live-streaming session on an e-commerce platform with the intent to make a purchase. The streaming room is hosted by two presenters (one male, one female) and has approximately 1,000 concurrent viewers. The hosts demonstrate products while engaging actively with their audience, and viewers share and communicate through live comments.

**Part II: Social Interaction Text Stimuli**

Note: This study employs a 2×2 design with Product Involvement (High vs. Low) and Interaction Type (Information vs. Relationship). Each participant will be randomly assigned to one of the four experimental conditions below.

***Condition 1: High Involvement-Information Interaction***

**Experiment 1 (Laptop):** You want to purchase a laptop computer and enter a live-streaming room selling "Tik" brand computers. The product is priced at 6,288 yuan, featuring a 16.9-inch screen, 20.0mm thickness, 1TB hard drive, 16GB memory, and R9-7940HX CPU. The hosts introduce detailed product specifications while viewers inquire about performance features and after-sales policies. Viewers compare specifications based on their personal needs and share their opinions.

**Experiment 2 (****Mobile phone)**: You want to purchase a mobile phone and enter a live-streaming room selling "Tik" brand phones. The product is priced at 4,888 yuan, featuring a 6.82-inch OLED curved screen, 12GB RAM, 512GB storage, and a 50-megapixel rear camera. The hosts provide detailed technical information while viewers ask specific questions about performance and warranty. Viewers engage in technical discussions, comparing features based on their usage requirements.

**Experiment 3 (Electric Bicycle):** You want to purchase an electric bicycle and enter a live-streaming room selling "Tik" brand e-bikes. The product is priced at 2,188 yuan, featuring an aluminum alloy frame, 14-inch high-carbon steel wheels, removable 48V battery, lithium battery rated power of 251-400W, net weight of 21-30kg, theoretical range of 51-60km, and front and rear disc brakes. The hosts introduce detailed specifications while viewers inquire about performance features and after-sales policies. Viewers compare technical specifications based on their personal transportation needs and share informed opinions about the product's capabilities.

***Condition 2: High Involvement-Relationship Interaction***

**Experiment 1 (Laptop)**: You want to purchase a laptop computer and enter a live-streaming room selling "Tik" brand computers priced at 6,288 yuan. The hosts emphasize their family-like relationship with fans and highlight the merchant's excellent reputation by showcasing positive customer reviews. Viewers indicate that the two hosts have good credibility, treat fans very kindly, and they have always enjoyed their streaming style, believing the hosts genuinely care about their fans. Some viewers also mention that the streaming platform has comprehensive functionality, is user-friendly, and has good credibility, expressing confidence that the platform would not engage in fraudulent practices.

**Experiment 2 (Mobile phone)**: You want to purchase a mobile phone and enter a live-streaming room selling "Tik" brand phones priced at 4,888 yuan. The hosts treat fans as family members and emphasize merchant credibility by showcasing positive customer reviews. Viewers indicate that the two hosts have good credibility, treat fans very kindly, and they have always enjoyed their streaming style, believing the hosts genuinely care about their fans. Some viewers also mention that the streaming platform has comprehensive functionality, is user-friendly, and has good credibility, expressing confidence that the platform would not engage in fraudulent practices.

**Experiment 3 (Electric Bicycle):** You want to purchase an electric bicycle and enter a live-streaming room selling "Tik" brand e-bikes priced at 2,188 yuan. The hosts treat fans as family members and showcase the merchant's excellent reputation through positive customer reviews. Viewers indicate that the two hosts have good credibility, treat fans very kindly, and they have always enjoyed their streaming style, believing the hosts genuinely care about their fans. Some viewers also mention that the streaming platform has comprehensive functionality, is user-friendly, and has good credibility, expressing confidence that the platform would not engage in fraudulent practices.

***Condition 3: Low Involvement-Information Interaction***

**Experiment 1 (Cookies)**: You want to purchase a box of cookies and enter a live-streaming room selling "Nog" brand cookies priced at 38 yuan. The hosts describe the product's nutritional content, pleasant texture, sweet flavor, and superior value compared to competing brands. Viewers inquire about practical matters such as shipping areas and delivery costs. Discussions focus on product attributes, with viewers sharing experiences about taste and customer service quality.

**Experiment 2 (Jeans):** You want to purchase jeans and enter a live-streaming room selling "Nog" brand jeans priced at 128 yuan. The hosts highlight the high-quality materials, durability, fashion design, and cost-effectiveness compared to similar products. Viewers ask about shipping policies and share practical experiences regarding the product's return and exchange services. The focus remains on functional product information and practical purchasing considerations.

**Experiment 3 (****Headphones):** You want to purchase headphones and enter a live-streaming room selling "Nog" brand headphones priced at 98 yuan. The hosts describe compatibility with Huawei, Apple, Xiaomi, Vivo, OPPO and other headphones, highlighting superior sound quality, Type-C charging interface, IPX5 waterproof rating, IP5X dustproof performance, trendy design with multiple color options, and cost-effectiveness compared to competing brands. Viewers inquire about shipping coverage and delivery costs, while others share experiences about sound quality and return/exchange services.

***Condition 4: Low Involvement-Relationship Interaction***

**Experiment 1 (Cookies):** You want to purchase a box of cookies and enter a live-streaming room selling "Nog" brand cookies priced at 38 yuan. The hosts treat fans as family and showcase merchant credibility through positive reviews. Viewers indicate that the two hosts have good credibility, treat fans very kindly, and they have always enjoyed their streaming style, believing the hosts genuinely care about their fans. Some viewers also mention that the streaming platform has comprehensive functionality, is user-friendly, and has good credibility, expressing confidence that the platform would not engage in fraudulent practices.

**Experiment 2 (Jeans):** You want to purchase jeans and enter a live-streaming room selling "Nog" brand jeans priced at 128 yuan. The hosts emphasize their family-like relationship with fans and merchant reputation through customer testimonials. Viewers indicate that the two hosts have good credibility, treat fans very kindly, and they have always enjoyed their streaming style, believing the hosts genuinely care about their fans. Some viewers also mention that the streaming platform has comprehensive functionality, is user-friendly, and has good credibility, expressing confidence that the platform would not engage in fraudulent practices.

**Experiment 3 (****Headphones):** You want to purchase headphones and enter a live-streaming room selling "Nog" brand headphones priced at 98 yuan. The two hosts express that they always treat their fans like family members and introduce the merchant's excellent reputation, displaying positive reviews from previous buyers. Viewers indicate that the two hosts have good credibility, treat fans very kindly, and they have always enjoyed their streaming style, believing the hosts genuinely care about their fans. Some viewers also mention that the streaming platform has comprehensive functionality, is user-friendly, and has good credibility, expressing confidence that the platform would not engage in fraudulent practices.

**Part Ⅲ: Basic Personal Information**

1. Gender

🗆 Men 🗆 Women

2. Age

🗆 Under 18 🗆 18–25 🗆 26–35 🗆 36–45🗆 Over 45

3. Educational level

🗆 High school/technical secondary school and below

🗆 College degree

🗆 Bachelor's degree

🗆 Master's degree and above

4. Years of watching live streaming

🗆 Less than half a year 🗆 Half a year to two years 🗆 Two years and above

5. Platforms online duration

🗆 less than 1 hour per day 🗆 1-2 hours per day 🗆 2-3 hours per day 🗆 More than 3 hours per day

Thank you for completing this survey.

# Appendix B. Measurement Items

| Variable | Items | Source |
| --- | --- | --- |
| Information Interaction | 1. I can easily obtain product-related information through the host's introduction. | （Zhou et al. 2019） |
|  | 2. I can better understand the product's functionality and features through the host's introduction. |  |
|  | 3.The product-related information introduced by the host is helpful to me. |  |
|  | 4.The information shared by other customers is helpful to me. |  |
| Relationship Interaction | 1.Communication with other customers in the live-streaming room makes me feel happy. | （Liu et al. 2018） |
|  | 2. Communication with others in the live-streaming room makes me feel that we have harmonious relationships. |  |
|  | 3.The host's emotions toward customers during the live-streaming are genuine. |  |
| Product Involvement | 1.You usually spend a lot of time making purchasing decisions for this type of product. | (Zaichkowsky, 1994;  Mulcahy et al.2020) |
|  | 2.When purchasing this type of product, you would carefully consider and comprehensively analyze product information. |  |
|  | 3.You are very active in collecting information about this type of product. |  |
|  | 4.You are very interested in information about this type of product. |  |
|  | 5.This type of product is important to you. |  |
| Customer Trust | 1.When watching live streams, I believe that the products provided by merchants have quality assurance. | (Fulmer and Dirks, 2018) |
|  | 2.When watching live streams, I have a high level of trust in merchants' resource integration capabilities. |  |
|  | 3.When watching live streams, I have confidence in merchants' credibility. |  |
| Cognitive Lock-In | 1.When watching live streams, I prioritize visiting followed live-streaming rooms and choose to purchase from them, even when I know they may not be the best option. | (Shi et al., 2018) |
|  | 2.When watching live streams, searching for and switching to other live-streaming rooms for shopping wastes the knowledge and skills I have already acquired. |  |
|  | 3.When watching live streams, searching for and switching to other live-streaming rooms for shopping can be very troublesome. |  |
|  | 4. When watching live streams, searching for and switching to other live-streaming rooms for shopping makes me feel like I'm wasting time and energy. |  |
| Utilitarian Motivation | 1.When shopping in live-streaming e-commerce, I mainly execute my pre-set plans. | (Büttner et al., 2014;  Voss et al. , 2003) |
|  | 2.When shopping in live-streaming e-commerce, I always hope to complete it as quickly as possible. |  |
|  | 3.When shopping in live-streaming e-commerce, my behavior is usually deliberate and goal-oriented. |  |
| Hedonic Motivation | 1.When shopping in live-streaming e-commerce, I frequently experience pleasure. |  |
|  | 2. For me, live-streaming e-commerce shopping is usually a way to seek entertainment. |  |
|  | 3. I like to use live-streaming e-commerce shopping to pass time. |  |
|  | 4.When shopping in live-streaming e-commerce, I enjoy browsing around. |  |
| Visit Stickiness | 1.I would prefer to stay in this live-streaming room for a long time in the future. | (Jiao et al., 2023) |
|  | 2.I will visit this live-streaming room very frequently in the future. |  |
|  | 3. I will follow the updates of this live-streaming room for a long time in the future. |  |
|  | 4.I will consistently maintain my habit of visiting this live-streaming room in the future. |  |
| Purchase Stickiness | 1.I will purchase products in this live-streaming room very frequently in the future. |  |
|  | 2.I will purchase many different types of products in this live-streaming room in the future. |  |
|  | 3.I will purchase a very large quantity of products in this live-streaming room in the future. |  |
|  | 4.I will consistently maintain my shopping habits in this live-streaming room in the future. |  |
